# Prolonged postnatal adaptation and enhanced prevalence of congenital heart diseases due to altitude may contribute to newborn mortality in Bolivia

**DOI:** 10.1113/EP092215

**Published:** 2025-03-11

**Authors:** Alexandra Heath, Inge von Alvensleben, Jesús Ardiles Spielvogel, Pablo Freudenthal, Johannes Trapp, Ivanna Noya, Miguel Gálvez, Fanny Mendizábal, Mariana Gonzales, Ceylan Apaza, Leibniz Sanga, Erin Mc Cann, Colleen G. Julian

**Affiliations:** ^1^ Department for Pediatric Cardiology Kardiozentrum La Paz Bolivia; ^2^ School of Medicine, Faculty of Health Witten/Herdecke University Germany; ^3^ Department of Mathematics School of Computation, Information and Technology, Technical University of Munich Garching Germany; ^4^ Department for Pediatric Cardiology Hospital ‘Arco Iris’ La Paz Bolivia; ^5^ Department of Pediatrics Hospital Municipal Boliviano Holandés El Alto Bolivia; ^6^ Department of Pediatrics University of Cincinnati Cincinnati Bolivia; ^7^ Department of Pediatrics University of Cincinnatti Cincinnatti USA

**Keywords:** cardiopulmonary transition, congenital heart disease, high altitude, hypoxia, oxygen dependency, pulmonary hypertension, sudden infant death

## Abstract

Highland populations suffer from significant infant mortality due to chronic ambient hypoxia, which increases the risk of congenital heart disease (CHD) and neonatal pulmonary hypertension. Neither the prevalence of these conditions nor the effectiveness of neonatal cardiac screening to identify CHD or pulmonary hypertension among neonates born at altitudes >4000 m in Bolivia has been reported. In a study of 1033 newborns in El Alto, Bolivia (4150 m), we determined the prevalence of CHD and prolonged postnatal adaptation. We also tested the accuracy of a neonatal cardiac screening tool in identifying infants with/without these conditions. Finally, diagnoses were contrasted between offspring born to parents of lowland versus highland origin. CHD was found in 54 neonates (5.2%), with the most common diagnoses being patent ductus arteriosus and atrial septal defect. Pulmonary hypertension without CHD was observed in 64 neonates (6.8%), with seven cases of persistent pulmonary hypertension of the newborn (PPHN). The neonatal cardiac screening tool showed a sensitivity of 45% and specificity of 99% for CHD, and 35% sensitivity and 92% specificity for prolonged pulmonary adaptation. Offspring of highland‐origin women tended to have increased CHD risk, while those from lower altitudes were predisposed to prolonged postnatal adaptation and PPHN; paternal altitude of origin had no statistic significance but showed same tendency. The high prevalence of relevant CHD and prolonged pulmonary adaptation in neonates born >4000 m in Bolivia likely contributes to the high infant mortality rates observed. The poor sensitivity of the pilot neonatal cardiac screening instrument underscores the need to develop evidence‐based tools optimized for use in low‐resource, high‐altitude settings.

## Abstract (highlights)

- **What is the central question of this study?**
  What are the incidence and distribution of congenital heart disease (CHD) in highland neonates and how effective is a neonatal cardiac screening (NCS) tool for early detection? The incidence of CHD was 5.2% and the distribution showed ductus arteriosus as the most frequent CHD. The NCS showed a specificity of 99% and a sensibility of 45% for CHD.
- **What is the main finding and its importance?**
  CHD incidence was more than 5 times higher than the global average, and some (6%) neonates exhibited hypoxaemia due to prolonged postnatal adaptation and pulmonary hypertension of the newborn. Early CHD diagnosis enabled timely and structured treatment, underscoring the need for a sensitive, user‐friendly NCS tool to detect CHD in highland newborns.

## 1 INTRODUCTION

Bolivia has the highest infant mortality rate in Latin America, mainly due to deaths occurring in the neonatal period (Burstein et al., 2019). For context, Bolivian infants have a 5.4‐fold greater probability of dying between birth and 1 year of age compared to infants born in the United Kingdom (19.6 deaths/1000 live births compared to 3.6 deaths/1000 live births) (WHO, 2022). Despite improved healthcare and healthcare access over the last few decades, infant mortality rates in the Bolivian highlands remain nearly double those reported for lowland areas of the country (WHO, 2022). While many factors contribute to these intra‐ and inter‐country differences, cardiopulmonary disorders and poor oxygenation are important contributors to the excessive infant mortality burden in the Bolivian highlands.

Nearly one‐third of Bolivians live above 3500 m (Tremblay & Ainslie, 2021). At this altitude, the partial pressure of inspired oxygen falls to approximately 60% of sea level values. Exposure to this level of hypobaric hypoxia immediately after birth can prohibit requisite ventilatory adaptations and delay the neonatal cardiopulmonary transition with potentially severe acute effects and long‐term sequelae for pulmonary vascular health. In highland neonates, the cardiopulmonary transition may be incomplete for weeks or months, and, in some cases, elevated pulmonary artery pressure persists (Gamboa & Marticorena, 1971; Niermeyer, 2003). Congenital heart disease (CHD) is also more common in neonates born at very high altitudes partly because decreased oxygen tension impedes spontaneous closure of the ductus arteriosus postnatally (Penaloza et al., 2008). As a result, the distribution of CHD differs from that observed at lower altitudes, with patent ductus arteriosus (PDA) prevalence being greater at high than low altitudes (Miao et al., 1988). Establishing the prevalence of delayed neonatal cardiopulmonary transition, pulmonary hypertension and CHD risk is a crucial first step in prioritizing areas of clinical need, given the high infant mortality rates in the Bolivian highlands.

Neonatal cardiac screening, including pulse oximetry, is standard practice in high‐income countries to identify infants with critical CHD (de‐Wahl Granelli et al., 2009; Garg et al., 2013; Martin et al., 2020; Plana et al., 2018). In contrast, neonatal cardiac screening is not broadly implemented in low‐ and middle‐income countries or low‐resource settings like Bolivia. Implementing a standardized neonatal cardiac screening protocol is challenging in Bolivia due to insufficient training resources and the absence of coordinated, electronic information systems for effective patient monitoring, follow‐up and referral. Adopting and executing a standardized neonatal cardiac screening protocol is crucial for the early identification and treatment of CHD, and its inclusion in the national norms for universal newborn screening protocols is urgently needed to reduce infant mortality rates. The Bolivian Ministry of Health has made advancements by releasing high‐altitude $S_{pO_{2}}$ standards for the referral of neonates to a paediatric cardiologist (Ministerio de Salud del Estado Plurinacional de Bolivia, 2019). However, evidence‐based $S_{pO_{2}}$ cut‐offs must be established at extremely high altitudes, given the lower oxygen saturations among neonates in the first 24 h of life.

The AJAYU project was designed to (1) establish the prevalence of CHD and pulmonary hypertension in neonates (*n* = 1033) born at extremely high altitudes (4150 m), in El Alto, Bolivia, and (2) evaluate the effectiveness of a simple neonatal cardiac screening instrument in identifying infants affected by CHD or prolonged pulmonary adaptation. In Aymara culture, *ayaju* is a nuanced term broadly referring to the ‘human spirit’. It is believed that people are composed of a physical body and a spiritual body, the latter consisting of three parts: the *jacha ajayu* (great spirit), the *jisja ajayu* (little spirit) and the *kamasa* (shadow) (Paredes, 1972). When a traumatic event such as an accident or the death of a child occurs, the *ajayu* is said to leave the body of those who are left mourning and, subsequently, it must be sought and retrieved (Estermann, 2019; Martínez‐Radl et al., 2023). While the AJAYU project focused on Bolivian highlanders, our findings may serve as a foundation for reducing neonatal morbidity and mortality due to cardiopulmonary disease in the expanding highland populations worldwide (Table 1).

**TABLE 1:** Recent expansion of populations living at extremely high altitudes (>3500 m).

City	Elevation (m)	Population 2000	Population 2020	Growth (%)
El Alto, Bolivia	4150	∼650,000	∼1,000,000	∼54%
La Paz, Bolivia	3869	∼1,600,000 (metro)	∼2,200,000 (metro)	∼38%
Lhasa, Tibet, China	3650	∼180,000	∼250,000	∼39%
Juliaca, Peru	3825	∼180,000	∼276,000	∼53%
Potosí, Bolivia	4090	∼140,000	∼240,000	∼71%
Oruro, Bolivia	3735	∼200,000	∼285,000	∼43%
Shigatse, Tibet, China	4000	∼80,000	∼100,000	∼25%
La Rinconada, Peru	5100	∼30,000	∼50,000	∼67%
Tingri, Nepal	4335	∼5000	∼7000	∼40%

*Source*: Instituto Nacional de Estadística de Bolivia, 2020; Instituto Nacional de Estadística e Informática Peru, 2020; National Bureau of Statistics of China, 2020; United Nations Department of Economic & Social Affairs, 2023; United Nations Economic Commission for Latin America & the Caribbean, 2021; World Bank, 2023.

## 2 METHODS

### 2.1 Ethical approval

Participants were enrolled following their written informed consent to study procedures conforming to standards set by the latest revision of the *Declaration of Helsinki* except for registration in a database, and approved by the Hospital Municipal Boliviano Holandés ethical board (Approval Number: J.E.I./HMBH/404/2018).

### 2.2 Study population and design

This observational study included a randomized sample of 1033 live infants born at Hospital Holandés (4150 m, El Alto, Bolivia) between December 2020 and January 2023 to establish a representative sample of maternal–infant pairs of similar ancestry and socioeconomic status living at extremely high altitudes. Our study cohort represents 20% of the 5156 infants born at Hospital Holandés during the study period.

Newborn and delivery records were reviewed by Kardiozentrum study personnel with the assistance of the on‐duty paediatrician to identify infants born on the randomized days that study personnel were at the hospital. No inclusion criteria, other than parental consent, were imposed to ensure the data were generalizable to the patient population. Only infants whose parents did not consent to participation were excluded.

After obtaining parental informed consent, participants completed a standardized questionnaire (SQ) to provide demographic information, parental altitude of birth and current residence, and familial and obstetric health history. Hospital staff then completed a clinical examination checklist or neonatal cardiac screening to record birth and newborn information, including pulse oximetry. For the neonate, the neonatal cardiac screening included evidence (yes/no) of breathing difficulty, external malformation, absence or presence of a pulse in all four extremities, arrhythmia, enlarged liver, hyperdynamic precordium, central cyanosis, prominent hemithorax, tachycardia, precordial thrill, heart murmur, and suspicion of cardiopathy or prolonged postnatal adaptation. $S_{pO_{2}}$ in the upper and lower right extremities (%) was also included. Infant sex (male, female, no data), mode of delivery (Caesarean section, vaginal, no data), and gestational age (GA) at delivery defined as term (between 37 and 42 weeks of pregnancy) or preterm (before 37 weeks of pregnancy) were recorded. Birth weight percentiles adjusted for GA and sex were classified as small for gestational age (SGA, below the 10th percentile), large for gestational age (LGA, above the 90th percentile), or appropriate‐for‐gestational age (AGA, between the 10th and 90th percentiles) using Ped(z). Maternal age at delivery was also recorded and defined as advanced for those aged 35 years or above at the time of delivery or adolescence for women aged 19 or less. Parental altitude of birth and childhood was categorized as a dichotomous variable: higher altitude (≥3000 m) or lower altitude (<3000 m). Obstetric history, including diagnoses of preeclampsia, gestational diabetes and diabetes (type I or II), was obtained from medical records for women with a prior pregnancy.

Our cardiac screening tool (https://linktr.ee/projectajayu) was adapted from standards provided by the Bolivian Ministry of Health for pulse oximetry and augmented to include clinical signs of cardiopulmonary compromise that are accepted criteria for CHD.

### 2.3 Echocardiography

Neonatal echocardiography studies, including standard cardiac structure and cardiopulmonary function measures, were performed by a paediatric cardiologist using an ESAOTE‐Cx echocardiograph with a neonatal 5 MHz transducer (Esaote, Genoa, Italy) as previously described (Heath‐Freudenthal et al., 2022). Echocardiography exams were performed within the first 6 days of life; the majority (66%) occurred within 24 and 48 h of life, with a median age of 35 h at the time of the study. Neonates were studied during wakefulness and in either the supine or lateral position. (1) Pulmonary hypertension was defined as a tricuspid regurgitation jet greater than 35 mmHg or a pulmonary artery acceleration time (PAAT) less than 80.2 ms (Koestenberger et al., 2017) and oxygen dependency with $S_{pO_{2}}$ less than 84%. (2) Persistent pulmonary hypertension of the newborn (PPHN) was defined as the failure of normal cardiovascular transition after birth, marked by pulmonary hypertension with hypoxaemia secondary to right‐to‐left shunting at the foramen ovale and ductus arteriosus; this often results in a higher pulmonary pressure compared with systemic pressure. Newborns were classified into five groups based on the presence and severity of echocardiographic observations (Table 2). Relevant CHD was defined as any malformation causing hypoxaemia, dilatation or hypertrophy of the cavities.

**TABLE 2:** Newborn echocardiographic classification.

Group	Category	Examples
Group 1	No abnormalities	None
Group 2	Haemodynamically irrelevant	e.g. Muscular VSDs or peripheral stenosis of the pulmonary arteries.
Group 3	Abnormal echocardiographic findings requiring further treatment or monitoring	e.g. Prolonged postnatal adaptation and relevant CHD for example, big ductus, ventricular and atrial septal defects, coartaction of the aorta
Group 4	Abnormalities requiring immediate attention	e.g. PPHN, tricuspidal atresia
Group 5	Abnormalities beyond the reach of treatment in Bolivia	e.g. Hypoplastic left heart syndrome

Abbreviations: PPHN, pulmonary hypertension of the newborn; VSD, ventricular septal defect.

Upper and lower right extremity $S_{pO_{2}}$ was also measured during the echocardiography exam using a Massimo neonatal pulse oximetry device (Massimo, Irvine, CA, USA) to obtain pre‐ and post‐ductal values. In neonates with $S_{pO_{2}}$ levels below 89% in both upper and lower extremities, a $S_{pO_{2}}$ value below 84% in either the upper or lower extremity or a differential $S_{pO_{2}}$ of >3% between the upper and lower extremities, pulse oximetry measurements were repeated after 1 h using technical norms released by the Bolivian Ministry of Health (Ministerio de Salud del Estado Plurinacional de Bolivia, 2019). Late postnatal adaptation was defined as neonatal hypoxaemia (a $S_{pO_{2}}$ < 84% at the repeated measurement) that could be managed with supplementary oxygen and occurred without CHD or severe pulmonary hypertension and right to left shunting, excluding PPHN).

### 2.4 Data analyses and management

Subject characteristics are shown as proportions for categorical variables or means (median) for continuous variables (Table 3). Accuracy parameters (sensitivity, specificity, positive predictive value and negative predictive value) were calculated to determine how well the NCS identified neonates with CHD or prolonged pulmonary adaptation. Frequencies were compared using the chi‐square test with a two‐sided *P*‐value <0.05 considered evidence of a difference in sample proportions. Data analyses and graphical representations were generated using GraphPad Prism v. 8.4.3 (GraphPad Software, Inc., San Diego, CA, USA). Data were stored in a secure digital database, and study identification numbers were assigned to each participant to preserve confidentiality.

**TABLE 3:** Subject characteristics (*n* = 1033).

Variable	Count
A. Newborn and delivery characteristics	
Infant sex	
Male	561 (54.31%)
Female	467 (45.21%)
No data	5 (0.48%)
Delivery type	
Caesarean	448 (43.37%)
Vaginal	560 (54.21%)
No data	25 (2.42%)
Term/preterm	
Term	926 (89.64%)
Premature	69 (6.68%)
No data	38 (3.68%)
Birth weight, all (g)	Average 3194 (median 3175)
Birth weight, full term (g)	Average 3231 (median 3215)
SGA, <10th	100 (9.68%)
LGA, >90th	34 (3.29%)
AGA, 10th–90th	600 (58.0%)
No data	299 (28.94%)
Newborn length (cm)	Average 50 (median 50)
B. Maternal characteristics	
Age (years)	Average 27.3 (median 27)
Advanced age (≥ 35 years)	178 (17.23%)
Young age (≤ 19 years)	138 (13.35%)
19–35 years	717 (69.42%)
Altitude of origin of the parents	
Over 3000 m	728 (70.47)
Below 3000 m	90 (8.71)
No data	215 (20.82)
Obstetric and health history	
Preeclampsia	119 (11.51)
Gestational diabetes	10 (0.96)
Diabetes, type I or II	6 (0.58)

Abbreviations: AGA, appropriate‐for‐gestational age; LGA, large for gestational age; SGA, small for gestational age

## 3 RESULTS

### 3.1 Cohort characteristics

As shown in Table 3, the study cohort included a similar proportion of male and female infants. Slightly over half of the infants were born vaginally, and the majority were born at term. While a small proportion (6.7%) were born preterm, no post‐term deliveries were observed in the study cohort. Birthweight across the study cohort averaged 3194 g. Of infants with complete birth weight, GA and sex information required to calculate birth weight percentiles, 81.7% were AGA, while 13.6% were SGA and 4.6% were LGA. Many infants (28.9%) were missing at least one data point required for birth weight percentile calculations.

Maternal age averaged 27 years, with a modest proportion of advanced maternal age or adolescent pregnancy (18% and 14%, respectively). Most women self‐identified as being of high‐altitude origin (Table 3). History of preeclampsia was common (11.5%), while diabetes (type I, II or gestational) was rare (<1%). Less than 10% of mothers or fathers were born and raised at low altitudes (8.7% and 9.5%, respectively).

### 3.2 Echocardiographic findings

#### 3.2.1 Distribution of CHD

In the entire cohort, 270 neonates had PDA (26.1%), and 54 (5.2%) had haemodynamically relevant CHD, including severe cases (0.5%). Of the 54 neonates with relevant CHD, the most frequent diagnoses were PDA (only haemodynamically relevant cases are included here, 35.2%), atrial septal defect (31.5%), coarctation or ‘narrowing’ of the aorta (11.1%), ventricular septal defect (VSD, 9.3%) and hypertrophic cardiomyopathy (3.7%). Hypertrophic cardiomyopathy was defined by an enlarged diastolic septal diameter (>2 SD) and observed in three patients. Rare conditions observed included tricuspid atresia, hypoplastic left heart, Ebstein's anomaly, transposition of the great arteries and cardiac tumour, with one case of each identified (9.3% of CHD, 0.5% of the total cohort).

**FIGURE 1:**
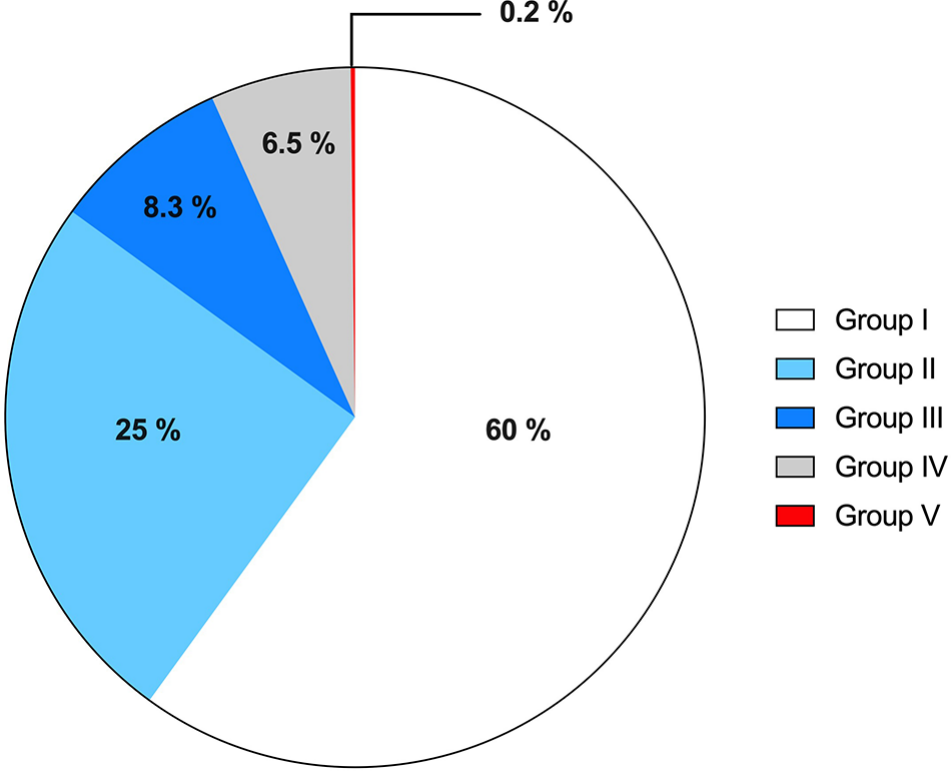
CHD classification: necessity of treatment according to echocardiographic findings. Most neonates showed no abnormalities (Group I, white) or haemodynamically irrelevant diagnoses (Group II, light blue) like muscular VSDs or peripheral stenosis of the pulmonary arteries. Group III (dark blue) presented a condition that may need further treatment at some point and was scheduled for follow‐up. Of neonates in Group IV (grey), few needed urgent cardiac surgery, while the majority were patients with low oxygen saturation and needed supplementary oxygen; these children had no cardiac malformation and were diagnosed with pulmonary hypertension. Finally, a small proportion (Group V, red) represented infants with conditions without possible treatment in Bolivia. CHD, relevant congenital heart disease; VSD, ventricular septal defect.

Based on echocardiographic findings, the cohort was catalogued into five groups (Table 2, Figure 1). Most neonates showed no abnormalities (Group I), or haemodynamically irrelevant diagnoses (Group II) like irrelevant neonatal ductus, muscular VSDs or peripheral stenosis of the pulmonary arteries. Group III presented a condition that may need further treatment, and the patient was scheduled for follow‐up visits. Of neonates in Group IV, few needed urgent cardiac surgery, while the majority were patients with low oxygen saturation and needed supplementary oxygen; these children had no cardiac malformation and were diagnosed with pulmonary hypertension. Finally, a small proportion (Group V) represented infants with conditions without possible treatment in Bolivia.

Pulmonary hypertension without relevant CHD (‘isolated pulmonary hypertension’) was observed in 64 patients (6.8%). Neonates with isolated pulmonary hypertension had reduced PAAT (72 ± 9 ms), while values averaged 61 ± 15 ms in the seven patients with PPHN; all neonates in the latter group were treated in the neonatal care unit.

**FIGURE 2:**
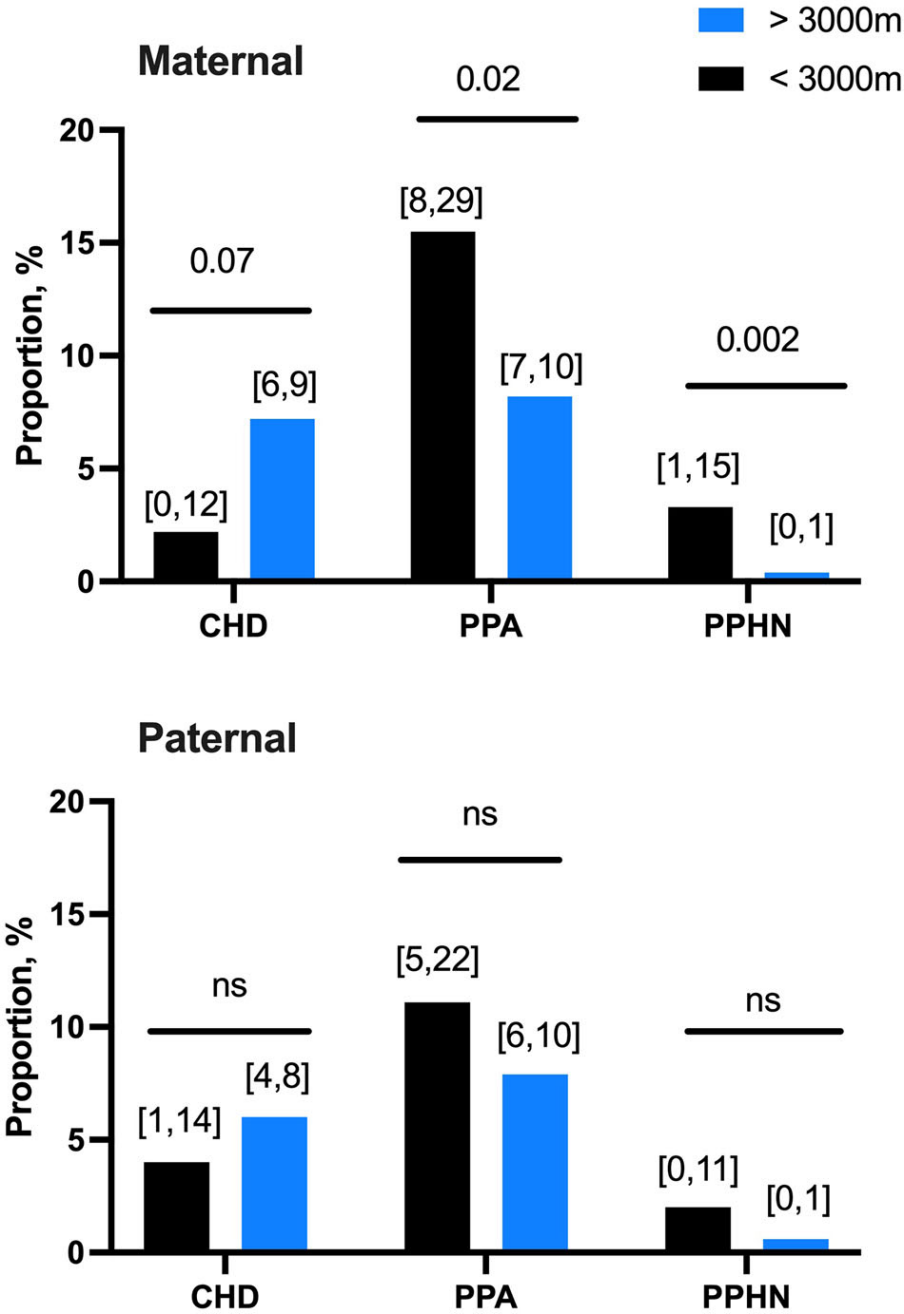
The proportion of neonates with CHD, delayed postnatal adaptation and persistent pulmonary hypertension among offspring of parents born and raised below 3000 m (black) or above 3000 m (blue). Chi‐squared tests were used to compare the proportions of each condition as a function of parental altitude of origin. *P*‐values for comparisons are shown. The 95% CI for each group is shown in brackets. Maternal <3000 m, *n* = 45; Maternal >3000 m *n* = 927; Paternal <3000 m, *n* = 48; Paternal >3000 m *n* = 683). CHD, congenital heart disease; PPA, prolonged postnatal adaptation; PPHN, persistent pulmonary hypertension of the newborn.

Offspring of women born and raised over an altitude of 3000 m tended to have an increased risk for CHD, whereas offspring of women from lower altitudes were predisposed to prolonged postnatal adaptation (*P* = 0.023) and PPHN (*P* = 0.002) (Figure 2). In contrast, the risk of CHD, prolonged pulmonary adaptation or PPHN did not differ by paternal altitude of birth (*P* > 0.05, all).

#### 3.2.2 Pulse oximetry screening and NCS checklist

In neonates without evidence of pulmonary hypertension or haemodynamically relevant CHD, upper and lower extremity $S_{pO_{2}}$ values averaged 90%, with the lower extremity having a larger range (78–98% vs. 84–99%). In contrast, neonates with CHD (cyanotic), PH or PPHN had comparatively reduced upper and lower extremity $S_{pO_{2}}$ values. Mean $S_{pO_{2}}$ and range for upper and lower extremities are as follows: CHD: 80% (75–82), 81% (77–83), PH: 83% (73–88) and 83% (71–89), PPHN: 76% (58–80) and 84% (68–89).

There was no difference in $S_{pO_{2}}$ (upper or lower) between infants born at term versus preterm (term: upper 90 (84–99) and lower 90 (80–98) vs. preterm: 91 (85–98) and 91 (85–98)). Infants born term and preterm also did not differ with respect to the proportion of infants diagnosed with PH (6.4% vs. 11.0%, *P* > 0.05), PPHN (1% vs. 0%, *P* > 0.05) or CHD (non‐cyanotic) (6% vs. 4%, *P* > 0.05). However, the proportion of preterm infants with cyanotic CHD (5.4%) was greater than that of term infants (0.002%; *P* < 0.001).

Using the standard questionnaire and NCS instrument, 33 out of 74 neonates with relevant CHD were correctly identified as positive, corresponding to a sensitivity of 45% (Table 4). Likewise, 25 out of 71 neonates with prolonged pulmonary adaptation were correctly identified as positive, corresponding to a sensitivity of 35%. In other words, the NCS correctly identified 45% of neonates with relevant CHD and 35% with prolonged pulmonary adaptation. Specificity, the ability of a test to correctly classify individuals who truly do not have the outcome of interest, was also calculated (Table 4). For CHD and prolonged pulmonary adaptation, the standard questionnaire and NCS instrument correctly identified 99% and 92% of neonates without CHD or prolonged pulmonary adaptation, respectively. Neonates with persistent low $S_{pO_{2}}$ without pulmonary hypertension or CHD were referred to a paediatric pulmonologist to rule out other pulmonary diseases.

**TABLE 4:** Accuracy of neonatal cardiac screening instrument.

A. CHD			
	CHD Diagnosis		
Suspicion	Yes	No	Total
Yes	33	8	41
No	41	861	902
Total	74	869	943

B. Prolonged pulmonary adaptation			
	PPA Diagnosis		
Suspicion	Yes	No	Total
Yes	25	77	102
No	46	877	923
Total	71	954	1025

*Note*: Cross tabulation showing the distribution of neonates suspected of CHD (A) or prolonged pulmonary adaptation (B) using our cardiac screening instrument (yes/no) compared to those who received a diagnosis (yes, no). Sensitivity, specificity, positive predictive value and negative predictive value for each diagnostic instrument are as follows: for CHD: sensitivity: 45; specificity: 99; PPV: 80; NPV: 95; for PPAt: sensitivity: 35; specificity: 92; PPV: 25; NPV: 95. Abbreviations: CHD, relevant congenital heart disease; NPV, negative predictive value; PPA, prolonged pulmonary adaptation; PPV, positive predictive value.

## 4 DISCUSSION

This project revealed a high prevalence of haemodynamically relevant CHD and prolonged pulmonary adaptation in neonates born at extreme altitudes in the Bolivian highlands. Based on our observations, 13% of newborns at 4150 m should be referred to a paediatric cardiologist. Prolonged pulmonary adaptation is particularly concerning in low‐resource, highland settings, given that infants born in these environments are often discharged from the hospital without having undergone neonatal cardiac screening or standardized pulse oximetry monitoring, which are standards of care in high‐income countries (Martin et al., 2020). Considering the high neonatal mortality rate in highlands, the elevation of pulmonary pressure and eventually pulmonary hypertension crisis could contribute to this hazard. The pilot neonatal cardiac screening instrument we implemented did not have high sensitivity for detecting neonates with CHD or prolonged pulmonary adaptation. However, nearly all infants who were suspected of having CHD or prolonged pulmonary adaptation using the instrument were diagnosed with the condition on echocardiographic exam. Our findings emphasize the need to develop a more sensitive, easy‐to‐use neonatal cardiac screening instrument for detecting CHD and prolonged pulmonary hypertension in newborns at high altitudes as well as the implementation of a training programme for nurses and medical staff.

Our findings, indicating that the prevalence of relevant CHD in highland neonates is at least five times higher than that reported worldwide (Hoffman et al., 2002), agree with prior observations made in geographically and ethnically diverse populations. In a cross‐sectional study of 84,302 school‐aged children living in Nagqu, Tibet (4500 m), a 5% CHD prevalence was also reported (Chun et al., 2019). In the Qinghai Province of China, a larger proportion (13%) of children aged 3–19 years living above 4200 m were diagnosed with CHD (He et al., 2022). Our observation that PDA (only those with left chamber dilatation) was common among highland neonates is in line with previous distribution reports. Like our findings, the most frequent paediatric CHD observed in Nagqu, Tibet, was PDA (66%), distantly followed by atrial septal defects (Chun et al., 2019). One study in Qinghai Province, China reported PDA to be the most common CHD in highland children (Miao et al., 1988), while a more recent study found atrial septal defects to be most prominent, followed by PDA and VSD (He et al., 2022). Using data obtained during annual CHD detection campaigns in Colombia, children living at moderate altitudes (1285–3000 m) had an increased incidence of PDA and left ventricular outflow obstruction diagnosis compared to children living at sea level, while the opposite was true for right ventricular outflow obstruction (Garcia et al., 2016); this demonstrates a unique CHD distribution even at modest altitudes. In neonates, PDA is quite common and often resolves without incident. However, close follow‐up is necessary until ductal closure is complete because persistent PDA left untreated may result in adverse outcomes, including heart failure, endocarditis or pulmonary oedema. In our cohort, atrial septal defects (with right chamber dilatation) were the second most common CHD, comprising a higher proportion of CHD cases than observed at sea level (Heath et al., 2012). While atrial septal defects may resolve within the first few months or years of life (Radzik et al., 1993), intervention may also be required (Shrivastava, 2000); this again highlights the need for close monitoring. Despite the high prevalence of CHD in the Bolivian highlands, Hospital Del Niño in La Paz is, to our knowledge, the only Bolivian public healthcare facility with the equipment and surgical capacity needed to both diagnose and treat children with CHD.

The prevalence of PPHN in our study cohort was 3.7‐fold greater than the global prevalence estimate of 1.8 cases per 1000 live births (Steurer et al., 2017). PPHN presents as severe hypoxaemia, high pulmonary vascular resistance, and right‐to‐left extrapulmonary shunting of deoxygenated blood in the absence of CHD (Mandell et al., 2021). Physiological abnormalities observed in PPHN include atypical pulmonary vasoreactivity, excessive muscularization of the pulmonary vasculature, and pulmonary hypoplasia (Mandell et al., 2021). Chronic environmental hypoxia has been shown to enhance medial thickness and muscularity of small pulmonary arteries and exaggerate postnatal pulmonary vascular contractile responses to hypoxia (Herrera et al., 2007, 2010). Insufficient oxygenation during perinatal life also results in irreversible alveolar simplification and slows the postnatal increase in lung volume in rats, which may lead to persistent hypoxia and, in turn, adverse pulmonary vascular outcomes (Blanco et al., 1991). Persistent pulmonary vasculature abnormalities at extreme altitudes may also be due to impaired pulmonary angiogenesis resulting from reduced bioavailability of the potent angiogenic protein vascular endothelial growth factor (VEGF) (Compernolle et al., 2002). In support of this, reduced VEGF expression was observed in infants with severe bronchopulmonary dysplasia, a condition often occurring alongside PPHN (Mourani et al., 2015). Animal models have also shown that maternal hypoxia reduced VEGF protein expression in the fetal lung (Mundo et al., 2021), and direct inhibition of VEGF in the fetal lung or amniotic fluid with sFlt1 (soluble fms‐like tyrosine kinase) or other VEGF‐specific antagonists led to pruning of the pulmonary vasculature and increased pulmonary arterial wall thickness (Le Cras et al., 2002; Tang et al., 2012). Future studies are required to improve understanding of the molecular and physiological mechanisms by which the hypoxia of high altitude increases the risk of PPHN, given the high mortality rates associated with the condition (up to 10% for severe cases) (Steurer et al., 2017) and the large proportion of surviving infants with long‐term morbidity (Konduri et al., 2007) even with appropriate therapeutic intervention.

In the present cohort, we also identified many hypoxic neonates in whom pulmonary hypertension, defined by reduced PAAT, flattening of the interventricular septum, or right ventricle hypertrophy without haemodynamically relevant CHD was detected. These neonates required supplementary oxygen but not intensive care; we therefore consider these infants to represent ‘prolonged postnatal adaptation’. It is well known that insufficient oxygenation immediately after birth due to high altitude hypoxia can interrupt requisite ventilatory adaptations and delay the neonatal cardiopulmonary transition with potentially severe acute effects and long‐term sequela for pulmonary vascular health (Niermeyer, 2003). In healthy, lowland‐born neonates, pulmonary vascular resistance and pressure decrease sharply with the onset of breathing at birth, increasing pulmonary blood flow and establishing the lungs as gas exchange organs. In contrast, the cardiopulmonary transition may be incomplete for weeks or months for highland neonates (Gamboa & Marticorena, 1971; Niermeyer, 2003).

Although prolonged pulmonary adaptation may appear more innocuous than PPHN, the combination of sustained neonatal hypoxaemia, rapid hospital discharge, and the absence of standardized pre‐ or post‐discharge pulse oximetry monitoring protocols creates an extremely dangerous scenario. Prolonged hypoxaemia is a frequent and well‐known trigger for sinus bradycardia and cardiac arrest in neonates. Hospital discharge before stabilization of $S_{pO_{2}}$, the lack of opportunity for remote pulse oximetry screening, and home birth likely contribute to the increased risk of sudden infant death observed in highland infants (Katz et al., 2015). As we reported elsewhere (Heath‐Freudenthal et al., 2024), most infants delivered vaginally at our study site are discharged 24 h after birth, well before the completion of pulmonary adaptation for many neonates born at high altitudes. The early recognition of oxygen dependency and adequate monitoring of hypoxaemic neonates can save lives. We therefore recommend that high‐altitude clinics and hospitals integrate a standardized neonatal pulse oximetry protocol as a standard of care, provide regular training opportunities for staff in the proper techniques and data interpretation, and encourage remote pulse oximetry screening and education where possible. Our prior clinical observations suggest infants receiving oral sildenafil alongside supplemental O_2_ may reach normal $S_{pO_{2}}$ levels more than 2 weeks earlier than their counterparts who received supplemental O_2_ alone (Heath‐Freudenthal et al., 2024); however, randomized controlled trials with longitudinal follow‐up are required to assess the efficacy and long‐term effects of such treatment in highland neonates before recommendations can be made.

The increased incidence of preeclampsia and fetal growth restriction observed at high altitudes (Bailey et al., 2022; Beall, 1981; Giussani et al., 2001; Gonzales & Tapia, 2009; Jensen & Moore, 1997; Julian et al., 2008, 2007; Krampl et al., 2001; Lichty et al., 1957; Mortola et al., 2000; Palmer et al., 1999; Soria et al., 2013; Yang et al., 2020; Zamudio et al., 1995) likely contributes to excess risk of CHD (Miao et al., 1988), neonatal pulmonary hypertension, and prolonged neonatal pulmonary adaptation by exaggerating fetal hypoxaemia and impairing pulmonary vascular development (Heath‐Freudenthal et al., 2022; Salinas‐Salmon et al., 2023). Notably, infant birthweight in the present study aligns with prior studies of highland Bolivians (Julian et al., 2007; Soria et al., 2013). It has been proposed that angiogenic imbalance contributes to pulmonary vascular abnormalities in the offspring of preeclamptic women at high altitudes (Heath‐Freudenthal et al., 2022; Mundo et al., 2021). In this study, prenatal visit records did not contain sufficient information to determine whether women did or did not have preeclampsia for the current pregnancy. Likewise, given that our primary objective was to describe the incidence and distribution of CHD, maternal arterial oxygenation ($P_{aO_{2}}$) was not obtained. Future studies should consider the effect of maternal hypoxia and vascular disorders of pregnancy on CHD and prolonged pulmonary adaptation in neonates born at extremely high altitudes.

Our study also highlights the challenges in implementing neonatal cardiac screening in low‐resource settings. Future research is needed to establish an effective, easy‐to‐use checklist for signs and symptoms of CHD in high‐altitude settings to improve the early detection and treatment of the condition. Supporting the successful implementation of NCS in low‐resource settings, a recent pilot study of 1592 infants at Muhimbili National Hospital in Tanzania identified 2.5 critical CHD cases per 1000 live births with a false positive rate of only 0.6% (Majani et al., 2022). Recent literature also highlights the need for ongoing evaluation of evidence‐based NCS protocols (Tsao et al., 2023). For example, referral criteria for neonatal critical CHD screening vary widely with different saturation thresholds, variable timing intervals for repeat $S_{pO_{2}}$ measurement, and the absence (or requirement) of including pre‐ and post‐ductal $S_{pO_{2}}$ measurements (Mahle et al., 2012; Riede et al., 2018; Singh et al., 2014; Thangaratinam et al., 2012). These differences raise concerns regarding the potential for increased false‐negative rates due to differences in neonatal cardiac screening instruments and highlight the need for altitude‐specific NCS instruments. While our tool was designed for use in low‐resource, high‐altitude settings, we acknowledge the need for improvements, particularly regarding its sensitivity in detecting CHD. One potential limitation of using pulse oximetry for neonatal cardiac screening is that current devices may underdiagnose the degree of hypoxaemia at extreme altitudes.

To accomplish these aims, a comprehensive evaluation of service infrastructure needs and consideration of alternative methods for clinical care for facilities without onsite pulse oximetry or echocardiography. In addition, collaboration with state databases and incorporation of neonatal cardiac screening data into the National Health Information System (SUIS) digital record could be highly relevant for the success of a CHD screening and treatment programme. Digital record‐keeping can more effectively manage the collection and analysis of data, improve quality control, and reduce the burden associated with manual data surveillance and reporting. Moreover, digital records could improve the referral and treatment process by ensuring accurate and quick access to patient data. Finally, integrating findings obtained via the neonatal cardiac screening into the SUIS could enable adjusting screening protocols based on real‐time data. We also consider that a national annual programme for CHD screening would further aid in identifying affected individuals.

### 4.1 Conclusions

The high prevalence of CHD and prolonged pulmonary adaptation in this cohort of highland neonates raises awareness about the enhanced risk of mortality and morbidity of children born at high altitudes. Pulmonary hypertension as part of a prolonged postnatal adaptation and its extreme presentation, the persistent fetal circulation, has an important representation in this cohort and may play a crucial role in sudden infant death; its recognition is likely to reduce newborn mortality in Bolivia. Our research also underscores the need to implement a standardized clinical examination protocol and proper pulse oximetry in every newborn and include such instruments in Bolivia's national norms for universal newborn screening protocols. However, a neonatal cardiac screening instrument optimized for high altitudes, particularly in low‐resource settings, is critically needed; our ongoing efforts focus on this need.

## AUTHOR CONTRIBUTIONS

Alexandra Heath, Inge von Alvensleben, and Colleen G. Julian conceived and designed the work; acquired, analyzed, and interpreted data; drafted the manuscript; and provided revisions for important intellectual content. Jesús Ardiles Spielvogel, Pablo Freudenthal, Johannes Trapp, Ivanna Noya, Miguel Gálvez, Fanny Mendizábal, Mariana Gonzales, Ceylan Apaza, Leibniz Sanga, and Erin Mc Cann acquired, analysed, and interpreted data and assisted with drafting sections of the manuscript. All authors approve of the final version of the manuscript and agree to be accountable for all aspects of the work in ensuring that questions related to the accuracy or integrity of any part of the work are appropriately investigated and resolved. All authors qualified for authorship and all those qualified for authorship are listed.

## CONFLICT OF INTEREST

None declared.

## Data Availability

Deidentified human subject data for subjects consenting to future data use is available from the corresponding author upon request.
